# Establishing a Zebrafish Functional Assay to Assess the Pathogenicity of Variants of Uncertain Significance in Ciliopathies

**DOI:** 10.1111/eci.70220

**Published:** 2026-05-11

**Authors:** Carla Aresi, Francesca Tonelli, Concetta Mazzotta, Valentina Serpieri, Cecilia Masiero, Camilla Torriani, Simona Villani, Enza Maria Valente, Antonella Forlino

**Affiliations:** ^1^ Department of Molecular Medicine, Biochemistry Unit University of Pavia Pavia Italy; ^2^ Department of Molecular Medicine, General Biology and Medical Genetics Unit University of Pavia Pavia Italy; ^3^ Department of Public Health and Experimental and Forensic Medicine, Unit of Biostatistics and Clinical Epidemiology University of Pavia Pavia Italy; ^4^ Neurogenetics Research Center IRCCS Mondino Foundation Pavia Italy; ^5^ Research Pyramid Program IRCCS Policlinico San Matteo Pavia Italy

**Keywords:** ciliopathies, morpholino, VUSs, zebrafish

## Abstract

A zebrafish morpholino knockdown model targeting *ahi1* enables efficient phenotypic assessment of ciliopathy‐related defects and functional evaluation of variants of uncertain significance. This assay clarifies the impact of VUSs, supporting zebrafish morphants as a reliable platform for validating ciliopathy‐associated genetic variants.

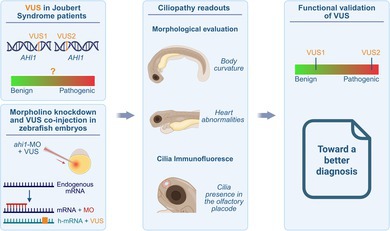

Reclassifying a “Variant of Uncertain Significance” VUS to pathogenic or benign remains a major challenge to improve the diagnostic yield in primary ciliopathies, and the development of functional assays to address this question is a pressing need. This study aims to fill this gap by assessing whether in vivo zebrafish‐based assays can help in reclassifying VUSs in ciliopathy genes. Primary ciliopathies comprise a heterogeneous group of autosomal or X‐linked recessive disorders caused by dysfunction of the non‐motile cilium, a single microtubule‐based organelle projecting from the surface of nearly all eukaryotic cells [1], which enables the detection of external stimuli and regulates key developmental processes [2]. Consequently, ciliary dysfunction leads to a wide spectrum of human diseases mainly affecting the central nervous system, retina, kidneys, liver, and skeletal system [3]. Despite the fact that over 100 genes responsible for primary ciliopathies are known, a subset of patients still remains undiagnosed, with negative consequences for management, prognosis, and family counselling. Such missing heritability often relates to the detection of VUSs that, based on current American College of Medical Genetics and Genomics (ACMG) criteria [4] and knowledge, can neither be classified as pathogenic/likely pathogenic nor as benign/likely benign.

The zebrafish is a well‐recognized vertebrate organism for studying heritable diseases including ciliopathies, and indeed several ciliopathy‐causative genes have been modelled both by morpholino (MO) knock‐down (KD) and by CRISPR/Cas9 or other genome‐editing knock‐out (KO) tools. For instance, among > 45 genes known to cause Joubert Syndrome (JS), the archetypal neurodevelopmental ciliopathy, more than half have at least one zebrafish model (morphant or mutant). These models commonly display organ‐specific ciliary dysfunctions [5] such as kidney cysts, retinal degeneration, body axis curvature, laterality defects and ventriculomegaly [6, 7, 8, 9, 10]. In this study, ciliopathy‐related phenotypes associated to loss‐of‐function mutations in three major JS‐causative genes, namely *AHI1, TMEM67* and *RPGRIP1L* (OMIM # 608894, 609884, 610937), were successfully reproduced by a MO approach (Figure 1A–C, Figure S1, Table S1). Although genome duplications are common in zebrafish, the three selected genes lack paralogs, supporting the specificity of a single MO in modulating the expression of the corresponding gene.

**FIGURE 1:**
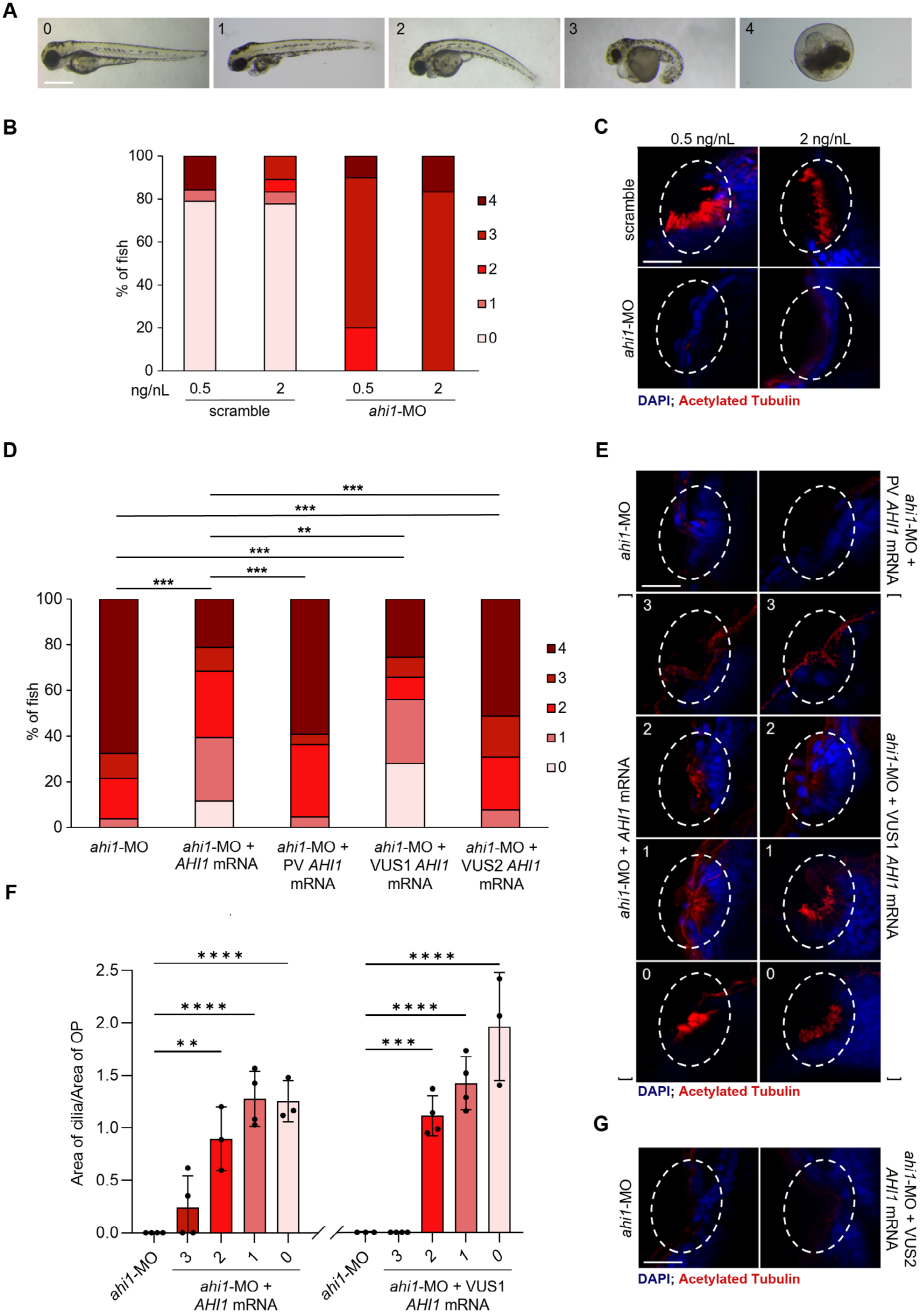
Establishing a morpholino‐based mRNA rescue platform to functionally classify ciliopathy VUSs in vivo. (A) Representative images of 3 dpf morphant severity categories (from 0 = normal to 4 = lethal), acquired by stereo microscopy at a 4× magnification. Scalebar: 500 μm. (B) Distribution of severity categories of *ahi1* morphants injected with two different MO concentrations (0.5 and 2 ng/nL). A scramble oligonucleotide was used as control using both selected doses. (C) Representative images of cilia area in olfactory placode (OP, dashed line) in morphants injected with scramble (*n* ≥ 3) and *ahi1*‐MO (*n* ≥ 4). Scalebar: 25 μm. (D) Distribution of severity categories of morphants injected with *ahi1*‐MO 0.5 ng/nL alone or co‐injected with *ahi1*‐MO and, respectively, 100 pg *AHI1* WT human mRNA, 100 pg *AHI1* pathogenic variant (PV), 100 pg *AHI1* VUS1 and 50 pg *AHI1* VUS2. (E) Representative cilia images for each category from severe (3) to normal (0) and (F) quantitation of cilia area in OP (dashed line) in morphants injected with *ahi1*‐MO alone (*n* = 10) or with *ahi1*‐MO and, respectively, 100 pg *AHI1* WT human mRNA (*n* = 14), 100 pg *AHI1* PV (*n* = 8), 100 pg *AHI1* VUS1 (*n* = 15). Scalebar: 25 μm. (G) Representative images of cilia area in OP (dashed line) in morphants injected with *ahi1*‐MO alone (*n* = 3) or co‐injected with *ahi1*‐MO and 50 pg *AHI1* VUS2 (*n* = 3). Scalebar: 25 μm. All the immunofluorescence images were acquired by confocal microscopy using water objective, magnification 25×, zoom 3×. Nuclei are stained with DAPI (in blue), cilia are stained by anti‐acetylated Tubulin (in red) (**p ≤ 0.01, ***p ≤ 0.001, ****p ≤ 0.0001).

*Ahi1*‐MO was then further exploited to establish a rapid, robust and easy phenotypic screening assay to facilitate the reclassification of VUSs based on the ability of constructs carrying these variants to rescue the ciliopathy phenotype. Early studies employing MO‐mediated KD of *ahi1* in zebrafish revealed severe developmental defects, including brain malformations, pronephric cysts, and disorganization of photoreceptors, consistent with a role for *ahi1* in ciliary structure and function [6]. More recent work using stable genetic KOs confirmed retinal involvement, showing progressive cone photoreceptor degeneration and variable phenotypic expressivity, but only at later age [11, 12]. The less severe outcome in KO fish compared to the morphants could be due to transcriptional compensation mechanisms [13]. Taking this into consideration, in order to develop a trustable model, a previously described MO targeting the splice junction between intron 12 and exon 13 [6] was selected for microinjection into zebrafish embryos, and the KD was proved by semiquantitative RT‐PCR (Figure S1A,B). Different MO concentrations (0.5 and 2 ng/nL) were tested to reproduce a ciliopathic but non‐lethal or too mild phenotype. A scramble MO was always injected as negative control. MO efficacy and toxicity are strictly dependent on injection parameters, particularly concentration. Given that scramble MOs can induce severe non‐specific phenotypes, parallel injections of gene‐specific and scramble control MOs using the same dose were systematically performed (Figure 1B, Figure S2A,B). This approach is essential to control procedural variables, including injection volume, embryo batch sensitivity, and needle consistency. To mitigate potential artefacts, MO concentrations were titrated to the lowest effective dose and validated against scramble injected siblings, ensuring that observed phenotypes resulted from targeted gene knockdown rather than off‐target effects or injection‐related toxicity. Embryonic development was monitored throughout the early stages, and at 3 days post‐fertilization (dpf), evaluating external morphological characteristics as well as cilia formation. Based on morphometric parameters, five severity categories were identified: normal development (0), straight body axis with heart abnormalities (1), curved body axis with abnormal heart (2), severe body deformity (3), and lethality (4) (Figure 1A). Two independent operators, blinded to the experimental conditions, performed all assessments by pure observation.

At both MO concentrations, 3 dpf *ahi1* morphants (*ahi1‐*MO) showed hydrocephalic head, abnormal body curvature, and heart bilateral symmetry, consistent with ciliopathy phenotypes (Figure 1B).

Cilia in the olfactory placode (OP), a paired embryonic ectodermal thickening that gives rise to the olfactory epithelium and related sensory structures [7], were detected by whole‐mount immunofluorescence staining with anti‐acetylated tubulin antibody (Figure S2C). A complete loss of cilia was evident in *ahi1*‐MO compared to controls (Figure 1C). The reproducibility and strength of the ciliopathy readout was further confirmed by knocking down two additional genes responsible for ciliopathies, namely *tmem67* and *rpgrip1l* (Supporting Information Results, Figure S2).

The establishment of a ciliopathy zebrafish model paved the way to standardize a MO‐based assay for functional assessment of the pathogenicity of VUSs, overcoming limitations such as genetic compensation and ethical constraints associated with the use of adult animals [13]. To this purpose, we selected the MO dosage of 0.5 ng/nL, resulting in the broadest distribution of phenotypic severity classes, minimal early lethality, and consistent reduction or loss of cilia in the olfactory placode. At this dose, embryos injected with the scramble MO showed no significant phenotypic differences compared with uninjected embryos. To confirm that the observed ciliopathy phenotype was caused by *ahi1* knock‐down, a rescue experiment was carried out by co‐injecting the *ahi1*‐MO (0.5 ng/nL) with in vitro synthesized wild‐type (WT) *AHI1* human mRNA (*AHI1* mRNA, NM_001134831.2, 100 pg) with the purpose to compensate the downregulation of the endogenous gene (Supporting Information Methods). Larvae were collected at 3 dpf and classified in the five severity categories described above. Co‐injection with WT *AHI1* mRNA significantly rescued the severe ciliopathy phenotype observed in *ahi1*‐MO morphants (*p* < 0.001). No effect was evident when WT *AHI1* mRNA was injected in control embryos or in *rpgrip1l* morphants (Figure S3A,B), supporting the specificity of the rescue experiment. The majority of co‐injected larvae fell into the less severe categories 0–2, showing a significantly decreased percentage of lethality (from 68% to 21%) (Figure 1D). Notably, the presence of cilia correlated with the severity of the observed phenotype. Similar to *ahi1*‐MO, the small subset of severely affected co‐injected larvae (category 3) exhibited a complete absence of cilia, while a progressive increase in ciliation was associated with milder phenotypic categories (Figure 1E). Indeed, the ciliary area was increased in co‐injected larvae characterized by the less severe phenotype (0–2) (Figure 1F). These findings confirmed that the WT *AHI1* mRNA significantly ameliorates the severe ciliopathic phenotype of *ahi1‐*MO.

To assess the suitability of *ahi1‐*MO to determine the pathogenic impact of missense variants, in vitro transcription of *AHI1* mRNA carrying the pathogenic variant (PV) c.2168G>A (p.R723Q) was performed. The mutant *AHI1* mRNA (100 pg) was co‐injected with *ahi1*‐MO into zebrafish embryos, and the severity compared to embryos injected only with *ahi1*‐MO and with both *ahi1*‐MO and WT *AHI1* mRNA, this latter referred to as the rescue group. The *ahi1*‐MO embryos co‐injected with the PV showed 59% lethality and a phenotypic distribution across the severity categories similar to the *ahi1*‐MO only injected embryos (Figure 1D), with complete loss of cilia formation in all the categories (Figure 1E). Moreover, a significant difference was evident between *ahi1‐*MO + PV and the rescue group (*p* < 0.001), for which the phenotypic distribution was more balanced and shifted towards the less severe categories. These results confirmed that a pathogenic human variant could not revert the phenotype in the *ahi*‐MO zebrafish, supporting the reliability of this assay. Next, we tested two *AHI1* VUSs: c.2273A>C (p.H758P, VUS1) and c.2009 T>C (p.L670P, VUS2) previously identified in patients with Joubert Syndrome. *In silico* analysis yielded conflicting predictions for VUS1 and possible deleterious consequences for VUS2, but were not sufficient to reliably classify either variant. Similarly to the co‐injection of WT mRNA with *ahi1*‐MO, the co‐injection of VUS1 mRNA revealed a significant difference in the distribution of the severity classes compared to *ahi1*‐MO only injected embryos, with a significantly greater proportion of larvae showing less severe phenotypes (categories 0 and 1) as well as a reduced percentage of early death (*p* < 0.001) (Figure 1D), supporting a benign outcome. In co‐injected embryos, a progressive and significant increase of the ciliated area was detected, in association with the milder phenotype (Figure 1E,F). Nevertheless, a conclusive assessment of no pathogenicity is challenging since a certain number of larvae were still classified in the lethal category (class 4). VUS2 *AHI1* mRNA was then co‐injected (100 pg) with *ahi1*‐MO. The 100% lethality detected at day 1 compared with the 68% caused by *ahi1‐*MO alone strongly suggested that this variant is detrimental. Embryos were then co‐injected with *ahi1*‐MO and a lower dose of VUS2 *AHI1* mRNA (50 pg). In these conditions, a high proportion of severe phenotypes were observed (51% lethality, 18% category 3, 23% category 2, and 8% category 1) (Figure 1D), along with a complete absence of cilia in the olfactory placode (Figure 1G). These results strongly supported the pathogenicity of VUS2 and went beyond the *in silico* severity prediction (Table S2).

This proof‐of‐principle study demonstrates the value of an in vivo zebrafish MO‐based functional assay for improving the interpretation of the pathogenic impact of VUS in ciliopathies, representing a reliable approach to overcome currently diagnostic challenges. In particular, the strength of this system lies in its ability to predict the pathogenic impact of a VUS when applied to sufficiently large sample sizes and coupled with robust, quantitative, and reproducible phenotypic readouts. The data strongly highlight the translational relevance of the zebrafish model and advocate for its systematic integration into clinical workflows.

## Author Contributions

**Carla Aresi and Francesca Tonelli:** conceptualization, data curation, formal analysis, investigation, methodology, project administration, validation, writing – original draft. **Concetta Mazzotta and Valentina Serpieri:** data curation, methodology, validation, writing – original draft. **Cecilia Masiero:** writing – review and editing. **Camilla Torriani and Simona Villani:** data curation, methodology, statistical analysis, validation, writing – original draft. **Enza Maria Valente:** conceptualization, data curation, investigation, project administration, writing – original draft, funding acquisition. **Antonella Forlino:** conceptualization, data curation, formal analysis, investigation, project administration, writing – original draft, funding acquisition.

## Funding

This work was supported by Ministero dell'Istruzione, dell'Università e della Ricerca, 2023‐2027, 20223C8C5B. Regione Lombardia, 3776/202000, Ministero della Salute, RF‐2019‐12369368, ERA‐NET, EUR002.

This work was supported by Italian Ministry of Education, University and Research (MIUR) [Dipartimenti di Eccellenza (2023‐2027)] to A.F.; by Regione Lombardia, “regionallawn 9/2020, resolutionn 3776/202000 to A.F.; by the European Union's—Next Generation EU” program through the Italian PRIN 2022—M4C2, Investimento 1.1—grant no. 20223C8C5B—CUP F53D23006870006 to A.F. Ricerca Finalizzata, Ministero della Salute, Bando 2019 RF‐2019‐12369368 to E.M.V. and A.F.; ERA‐NET Neuron NDCil project EUR002 to EMV.

## Conflicts of Interest

The authors declare no conflicts of interest.

## Supporting information


**Figure S1:** *ahi1*, *tmem67* and *rpgrip1l* downregulation. (A) *ahi1*, *tmem67* and *rpgrip1l* MOs target sites. (B‐C) To determine the molecular effect of morpholino (MO) knock down, the expression of *ahi1* and *tmem67* was evaluated in the RNA extracted from 2 dpf zebrafish control and morphants. (B) The splice‐blocking *ahi1*‐MO (0.5 ng/nL) targets the junction region between intron 12 and exon 13 of zebrafish *ahi1*, generating exon skipping and resulting in a 354 bp deletion [5]. RT‐PCR amplification products showed two bands of, respectively, 741 bp and 387 bp. Based on band intensity quantitation the semiquantitative RT‐PCR showed that 34.2% of the whole *ahi1* transcript was truncated, resulting in a shorter 354 bp amplicon (C) Splice‐blocking *tmem67*‐MO (2 ng/nL) targets the splice acceptor site between intron 8 and exon 9 [2]. qPCR analysis allowed to detect 34.5% reduction of *tmem67* expression in injected embryos compared to controls.
**Figure S2:** Overview of the zebrafish olfactory placode (OP) and *tmem67* and *rpgrip1l* morphants characterization. (A) Distribution of severity categories of *tmem67* morphants injected with two different MO concentrations (2 ng/nL and 3 ng/nL). A scramble oligonucleotide was used as control, and it was injected at all MO tested doses. (B) Distribution of severity categories of *rpgrip1l* morphants injected with four different concentrations (0.5 ng/nL, 1.25 ng/nL, 2.5 ng/nL and 5 ng/nL). A scramble oligonucleotide was used as control, and it was injected at all MO tested doses. (C) Scheme of a zebrafish scramble olfactory placode (OP). (i) Representative bright‐field image of zebrafish OP, Magnification 25×, zoom 1. Scalebar: 150 μm. (ii) Representative whole mount immunofluorescence image of cilia in the OP stained with anti‐acetylated tubulin antibody. Magnification 25×, zoom 1. Scalebar: 150 μm. (iii) Representative image of whole mount immunofluorescence of cilia in the OP, cilia stained with anti‐acetylated tubulin antibody. Magnification 25×, zoom 3×. Scalebar: 50 μm. (D) Representative images of cilia area in OP (dashed line) in morphants injected with scramble (*n* ≥ 4), *rpgrip1l*‐MO (*n* ≥ 6) and *tmem67*‐MO (*n* ≥ 11) for each concentration. Magnification 25×, zoom 3×. Scalebar: 25 μm. All the immunofluorescence images were acquired by confocal microscopy with water objective. Nuclei were stained with DAPI (in blue), cilia were labelled by anti‐ acetylated Tubulin (in red). (E) Quantitation of cilia area in OP in *tmem67* morphants compared to scramble control at all concentrations. (**p ≤ 0.01, ***p ≤ 0.001, ****p ≤ 0.0001).
**Figure S3:** Specificity of rescue. (A) Distribution of severity categories of *AHI1* WT mRNA (100 pg) injected embryos compared to not injected control. (B) Distribution of severity categories of *rpgrip1l* morphants (0.5 ng/nL) coinjected with *AHI1* WT mRNA (100 pg). Embryos injected only with *rpgrip1l*‐MO (0.5 ng/nL) were used as control.
**Table S1:** Number of samples per experiment.
**Table S2:** *In silico* predictions of selected variants. Variants pathogenicity was evaluated using the dbNSFP v5.0 (https://www.dbnsfp.org) [7], a widely used database developed for functional prediction and annotation of all potential non‐synonymous single‐nucleotide variants (nsSNVs) in the human genome. It reports pathogenicity based on prediction scores from several algorithms.

## Data Availability

The data that support the findings of this study are available from the corresponding author upon reasonable request.
