# Supplementary material for: Establishing a Zebrafish Functional Assay to Assess the Pathogenicity of Variants of Uncertain Significance in Ciliopathies
Source: Eur J Clin Invest. 2026 May 11;56:e70220. doi: 10.1111/eci.70220 (PMC13161512; doi:10.1111/eci.70220)
Supplement: Supplementary file 1 — Figure S1: ahi1, tmem67 and rpgrip1l downregulation. (A) ahi1, tmem67 and rpgrip1l MOs target sites. (B‐C) To determine the molecular effect of morpholino (MO) knock down, the expression of ahi1 and tmem67 was evaluated in the RNA extracted from 2 dpf zebrafish control and morphants. (B) The splice‐blocking ahi1‐MO (0.5 ng/nL) targets the junction region between intron 12 and exon 13 of zebrafish ahi1, generating exon skipping and resulting in a 354 bp deletion [5]. RT‐PCR amplification products showed two bands of, respectively, 741 bp and 387 bp. Based on band intensity quantitation the semiquantitative RT‐PCR showed that 34.2% of the whole ahi1 transcript was truncated, resulting in a shorter 354 bp amplicon (C) Splice‐blocking tmem67‐MO (2 ng/nL) targets the splice acceptor site between intron 8 and exon 9 [2]. qPCR analysis allowed to detect 34.5% reduction of tmem67 expression in injected embryos compared to controls. Figure S2: Overview of the zebrafish olfactory placode (OP) and tmem67 and rpgrip1l morphants characterization. (A) Distribution of severity categories of tmem67 morphants injected with two different MO concentrations (2 ng/nL and 3 ng/nL). A scramble oligonucleotide was used as control, and it was injected at all MO tested doses. (B) Distribution of severity categories of rpgrip1l morphants injected with four different concentrations (0.5 ng/nL, 1.25 ng/nL, 2.5 ng/nL and 5 ng/nL). A scramble oligonucleotide was used as control, and it was injected at all MO tested doses. (C) Scheme of a zebrafish scramble olfactory placode (OP). (i) Representative bright‐field image of zebrafish OP, Magnification 25×, zoom 1. Scalebar: 150 μm. (ii) Representative whole mount immunofluorescence image of cilia in the OP stained with anti‐acetylated tubulin antibody. Magnification 25×, zoom 1. Scalebar: 150 μm. (iii) Representative image of whole mount immunofluorescence of cilia in the OP, cilia stained with anti‐acetylated tubulin antibody. Magnification 25×, zoo [file ECI-56-e70220-s001.docx]

**Establishing a zebrafish functional assay to assess the pathogenicity of Variants of Uncertain Significance in Ciliopathies.**

Carla Aresi^1,*^, Francesca Tonelli^1, *^, Concetta Mazzotta^2^, Valentina Serpieri^2^, Cecilia Masiero^1^, Camilla Torriani^3^, Simona Villani^3^, Enza Maria Valente^2,4^, Antonella Forlino^1,5, #^

^1^ Department of Molecular Medicine, Biochemistry Unit, University of Pavia, Pavia, Italy

^2^ Department of Molecular Medicine, General Biology and Medical Genetics Unit, University of Pavia, Pavia, Italy

^3^ Department of Public Health and Experimental and Forensic Medicine, Unit of Biostatistics and Clinical Epidemiology, University of Pavia, Pavia, Italy

^4^ Neurogenetics Research Center, IRCCS Mondino Foundation, Pavia, Italy

^5^ Research Pyramid Program IRCCS Policlinico San Matteo, Pavia, Italy,

**SUPPLEMENTARY INFORMATION**

1. **MATERIALS AND METHODS**

## **Husbandry**

Wild-type (WT) AB zebrafish were obtained by the European Zebrafish Research Center (EZRC) (Germany). Zebrafish embryos were kept in petri dishes in zebrafish water (1.2 mM NaHCO_3_, instant ocean 0.1 g/L, 1.4 mM CaSO_4_, methylene blue 0.00002% w/v) at 28°C with 14/10 light/dark cycle. For the experiments, larvae were anesthetized using 0.016% w/v tricaine (3-amino benzoic acidethylester, Merck) in zebrafish water and sacrificed by tricaine overdose (0.03% w/v). All the experiments were performed in agreement with EU Directive 2010/63/EU.

**Embryo injections**

Microinjection was carried out using an InjectManmicromanipulator (Eppendorf) assembled on a Leica M165 FC stereomicroscope. Injection parameters were set to a pressure of 150 hPa and an injection time of 0.8 s. Three morpholinos (MOs) were used: splice MO (5’-CCACACTCTGAAAGGGAAAAACATT-3’) that targets the junction of intron 12 and exon 13 (I12E13) of zebrafish *ahi1* (ENSDARG00000044056, ZDB-MRPHLNO-120314-2) [1]; splice MO (5’-AGAACAACTACAGAAGAACAATAAC-3’), that targets the intron 8/exon 9 splice acceptor site of zebrafish *tmem67* (ENSDARG00000076752, ZDB-MRPHLNO-130603-2) [2]; translation blocking MO (5’-AGTTTCATCAGCACGAGAAAACATC-3’), that targets 1–24 bp downstream the translation start site of zebrafish *rpgrip1l* (ENSDARG00000051754) [3]. A scramble oligonucleotide (5’- CCTCTTACCTCAGTTACAATTTATA-3’) was injected as control at the same concentration of MOs to monitor the phenotypic consequence of RNA injection. MO stock 1 mM (Gene Tools) was diluted in Danieau solution (58 mM NaCl, 0.7 mM KCl, 0.4 mM MgSO_4_, 0.6 mM Ca(NO_3_)_2_, 5 mM Hepes, pH 7.6) containing a tracer dye (0.5 mg/mL, dextran conjugated with tetramethyl rhodamine (Molecular Probes). Different doses of MO were pre-heated at 65 °C for 10 min and then loaded in a glass needle in a final volume of 5 uL and 2-4 nL were injected into fertilized eggs at the stage of 1-2 cells. The day after, embryos were screened under a stereomicroscope Leica M165 FC connected to a Leica Flexacam C3 digital camera using a fluorescent DSred filter. Embryos showing a diffuse fluorescent signal in the body were considered properly injected and kept for the experiments. Lethality was evaluated at 3 days post fertilization (dpf) by counting surviving larvae, then morphological parameters were evaluated as detailed below. Number of injections performed: *ahi1:* n=3 for each concentration; *tmem67* and *rpgrip1l*: n=1 for each concentration; for rescue experiments n=4; PV: n=2, VUS1: n=4, VUS 2: n=3 using 100 pg and n=2 using 50 pg.

**Morphological analysis**

At 3 dpf, MO injected larvae were anesthetized with 0.016% w/v Tricaine (3-amino benzoic acidethylester, Merck) in zebrafish water and images were acquired under stereomicroscope Leica M165 FC connected to a Leica Flexacam C3 digital camera to evaluate morphants’ morphological features, based on five categories: “0” when anatomical structures were entirely normal for developmental stage; “1” if there was a single structural abnormality (e.g. abnormal heart bilateral simmetry); “2” if the morphant displayed more dysmorphic features together with structural abnormalities (e.g. hydrocephalic head, abnormal body curvature and abnormal heart bilateral symmetry); “3” if the morphant was completely deformed, and “4” for the lethal phenotype (**Fig. 1A**). Biological triplicates were performed for *ahi1*-MO generation, rescue experiment, VUS1 and VUS2 evaluation.

**Immunofluorescence for the characterization of cilia at the level of olfactory placode (OP)**

Injected larvae were fixed at 3 dpf in 4 % PFA in PBS for 2 h at room temperature (RT), washed in PBS, dehydrated in increasing series of 25%, 50% and 75% MeOH/PBST (PBS + 0.5 % Tween solution) and stored at -20°C in 100% methanol. Larvae were then rehydrated in decreasing series of 75%, 50% and 25% MeOH/PBST (PBS + 0.5 % Tween solution) 5 min each, permeabilized in pre-chilled acetone for 7 min, washed in ddH_2_O for 5 min followed by 5 min in PBDT (PBST + 1% DMSO) and equilibrated in blocking buffer (10 % BSA, 1% DMSO in PBST) for 2 h at RT. Samples were incubated with mouse anti-Acetylated tubulin at a dilution of 1:400 (T6793, Sigma-Aldrich, RRID:AB_477585) in blocking buffer solution overnight at 4° C. Then, samples were incubated with anti-mouse Alexa 647 (4410S, Cell signalling) secondary antibody at a 1:500 dilution in 2% BSA, 1% DMSO in PBST for 2 h at RT. Finally, larvae were incubated with 4′,6-diamidino-2-phenylindole (DAPI, Sigma-Aldrich) for 10 min. Each sample was mounted in 3% Metylcellulose and images were acquired by confocal microscopy Leica TCS SP5 DLS, 25x water objective, zoom 3. 2D images were generated from the z-stack using maximum intensity projection. Quantification of the area of the cilia in the olfactory placode was measured using Fiji (Image J) software [4]. For the MO characterization the number of stained larvae was: for the *ahi1-*MO experiments *ahi1-*MO ≥ 4 and scramble ≥ 3*, for tmem67*-MO experiments *tmem67*-MO ≥11 and scramble ≥5 and for *rpgrip1l*-MO experiments *rpgrip1l-*MO ≥6 and scramble ≥ 4. For rescue experiments the number of stained larvae was for the *ahi1-*MO experiments *ahi1-*MO= 3 and *ahi1-*MO *+ AHI1* WT mRNA = 14*.* For VUS1 and PV experiments the number of stained larvae was for the *ahi1-*MO = 7, *ahi1-*MO + PV = 8*, ahi1-*MO *+* VUS1 = 15; for VUS2 experiments the number of stained larvae was for the *ahi1-*MO = 3 and *ahi1-*MO *+* VUS2 =3.

**RNA extraction**

RNA was extracted from three pools of 2 dpf (n=10 embryos per pool) not injected and injected embryos using QIAzol Lysis Reagent (Qiagen) according to manufacturer’s instructions. DNA free kit DNase Treatment & Removal (Invitrogen) was used to eliminate genomic DNA traces. RNA quantity was determined by NanoDrop spectrophotometer, and its quality by agarose gel 1% w/v electrophoresis in TBE 1X buffer. Reverse-transcription was performed using the High-Capacity cDNA Transcription kit (Applied Biosystems) according to manufacturer’s protocol in a final volume of 20 μL.

The expression of *ahi1* was evaluated by semiquantitative PCR using the following primers: forward (5’- AGATGGGCTGTTTTACTCTC-3’) on exon 11 and reverse (5’-TTCCGCAAGGAGTGAACGTA-3’) on exon 16 [5]. *βactin* was used for normalization. PCR was performed in triplicate in 25 µL final volume (50 ng cDNA) using the following cycle: 2 min at 95 °C, 44 cycles of 5 s at 95 °C, 30 s at the 60°C followed by 1 s at 72 °C, and 10 min at 72 °C for final extension [6]. Following electrophoresis, equal volumes of PCR products were visualized on 1% agarose gel. Bands were quantified using Fiji (Image J) software.

The expression of *tmem67* was evaluated by real time quantitative PCR (qRT-PCR) using the following primers: forward (5’-GTCCAGTCAGCATGGTTTTCAA-3’) on exon 7 and reverse (5’- GTTTCCCAGAGACTGACATGC -3’) on exon 8. *loopern4* was used for normalization. qPCR was performed with SYBR Green Master mix (Applied Biosystems) in biological triplicate in 25 µL final volume (175 ng cDNA) using the following cycle: 2 min at 95 °C, 44 cycles of 5 s at 95 °C, 30 s at the 64°C followed by 1 s at 72 °C [6]. The dissociation curve was performed to confirm the specificity of the amplification. The relative expression was calculated using ΔΔCt method. The QuantStudio 3 thermocycler and the QuantStudio Design & Analysis software (Applied Biosystems) were used.

**Generation of *AHI1* transcripts**

To facilitate human *AHI1* gene cloning, a BamHI restriction site overhang was added to the 5′ end of the forward primer (5′-CCGGATCCATGCCTACAGCTGAGAGTGAAGC-3′), and a SpeI restriction site overhang was added to the 3′ end of the reverse primer (5′-GCCGACTAGTTTACTCTATTAGAGTGACTTTTCTG-3′). The human *AHI1* (RefSeq: NM_001134830.2) was amplified from the VB210811-1162ayc vector using Phusion High-Fidelity DNA Polymerase (Thermo Scientific). VB210811-1162ayc (9426 bp) was designed using VectorBuilder ([https://en.vectorbuilder.com](https://en.vectorbuilder.com)) containing a CAG promoter, the human *AHI1* canonical transcript (NM_001134830.2) tagged with V5 (N-terminus) and FLAG (C-terminus), and an ampicillin resistance gene for bacterial selection. PCR was performed using the following cycle: 30 s at 98 °C, followed by 35 cycles of 10 s at 98 °C and 1.5 min at the 68°C, lastly 10 min extension at 72 °C was applied. The amplicon, digested with BamHI and SpeI (Promega) 10 U/μL, was gel purified with Nucleospin Gel and PCR Clean-up kit (Machinery-Nagel) and cloned in p-Bluescript sk (+) (Life Science). Sanger sequencing was performed by Eurofins Genomics.

The resulting construct was linearized with SpeI (Promega) and purified using the Nucleospin Gel and PCR Clean-up Kit (Machinery-Nagel). DNA was transcribed using MEGAscript T7 Kit (Invitrogen). DNA was removed using Turbo DNA-*free* Kit (Life Technologies), polyadenylation of RNA was performed with Poly(A) Tailing Kit (Ambion) and transcript was purified using RNeasy Minielute Clean-up Kit (Qiagen). The *in vitro* transcribed mRNA was quantified by nanodrop and its quality was checked by electrophoresis on denaturing 1% MOPS/formaldehyde agarose gel and 1% Agarose in TBE.

The generation of the three *AHI1* transcripts, containing respectively the pathological variant (c.2168 G>A) and the two VUS (c.2273 A>C and c.2009 T>C), was performed as described above, starting from the amplification of *AHI1* (NM_001134830.2).

Briefly, for the c.2168 G>A variant a 967 bp sequence from *AHI1* containing the nucleotide change was synthesized by Eurofins Genomics. EcoRI and HindIII specific sites overhang were added at 5’ and 3’ primer extremities, respectively (forward primer 5’-GCGGCGAAGCTTCTGGGAGCCAATGGAAAT-3’; reverse primer 5’-GCGGCGGAATTCCCTTAAACTCAGTTTCTTT-3’). PCR was then applied to amplify the synthetic sequence using the following cycle: 30 s at 98 °C, followed by 35 cycles of 10 s at 98 °C, 30 s at 61°C and 30 s at 72°C, lastly 10 min extension at 72 °C. The amplicon was digested with EcoRI and HindIII (Promega) 10 U/μL, purified with Nucleospin Gel and PCR Clean-up kit (Machinery-Nagel) and cloned in p-Bluescript sk (+) (Life Science) containing *AHI1* (NM_001134830.2). Following plasmid transformation into JM109 competent cells (Thermo Scientific), positive colonies were selected and verified by sequencing (Eurofins Genomics). The c.2273A>C and c.2009T>C missense variants were introduced into the VB210811-1162ayc expression vector using the In-Fusion® HD Cloning Kit (TaKaRa Bio). The cloning strategy followed a classical restriction enzyme–based approach: the fragment of interest was amplified by high-fidelity PCR and replaced into the vector backbone. AccI and EcoRI restriction sites were used for digestion and ligation. Following plasmid transformation into DH10B competent E. coli cells, positive colonies were selected and verified by sequencing (Eurofins Genomics).

**mRNA Variants injection in MO-injected Embryos**

Phenotypic rescue was performed by co-injecting 100 pg of *in vitro* transcribed WT human *AHI1* mRNA along with *ahi1*-MO. The same amount of mRNA was used to test the pathogenic variant (PV) c.2168 G>A and the two VUS, c.2273 A>C (VUS1) and c.2009 T>C (VUS2). For the VUS2 a lower amount of mRNA (50 pg) was also used, as the first dose was lethal in our model.

**Statistical analysis**

Morphological analyses were conducted to characterize the distribution of severity scores and other categorical variables across experimental groups. Severity was evaluated on an ordinal scale from 0 (no observable phenotype) to 4 (severe phenotype). Results were summarized as counts and percentages for each severity level, and, when appropriate, measures of central tendency and dispersion (medians and interquartile ranges) were also reported to capture the distribution of ordinal data. To assess differences between groups, non-parametric statistical tests were applied, given the ordinal nature of the outcome variable. Categorical variables were summarized using absolute frequencies and percentages. Differences in the distribution of severity classes across treatment groups were assessed using Fisher’s exact test, which was consistently applied due to the presence of contingency tables with low expected frequencies. When the overall test was statistically significant, post-hoc pairwise comparisons were performed using Fisher’s exact test with Holm correction for multiple comparisons. Single comparisons between two independent groups were carried out with Unpaired t-test, to evaluate the area of cilia one-way ANOVA followed by Tuckey’s post hoc test was used. Statistical significance was set at p < 0.05. Analyses were conducted using R statistical software (version 4.4.1) and GraphPad Prism 9.3.1.

**RESULTS**

**Generation and characterization of *tmem67* and *rpgrip1l* morphants**

The knock down of *tmem67* and *rpgrip1l* both responsible for ciliopathies was tested to verify the reproducibility and strength of the MO ciliopathy characterization readout.

Previously published MOs were selected for microinjection into zebrafish embryos at the 2-cell stage testing different concentrations to have a broad distribution among the different categories (**Fig. S1A**). The effect of MO on gene expression was evaluated for *tmem67-*MO by qPCR **(Fig. S1C**), but not for *rpgrip1l*-MO, as this prevents the mRNA translation by targeting a region close to the translation starting site (Mahuzier et al., 2012). In a MO concentration-dependent manner, 3 dpf *tmem67* and *rpgrip1l* morphants showed hydrocephalic head, abnormal body curvature and heart bilateral symmetry, consistent with ciliopathy phenotypes (**Fig. S2A,B**). In particular, the two concentrations tested for *tmem67* (2 ng/nL and 3 ng/nL), revealed a significantly different distribution of the larvae in the categories. At 3 ng/nL, most larvae (52%) were lethal (category 4), while only 1.7% showed normal development (category 0). A straight body axis with heart abnormalities (category 1) was observed in 26% of larvae, while 14% of larvae were severely deformed (category 3). In contrast, at 2 ng/nL, 13% of the injected larvae had no phenotype (category 0), the majority of them (67%) fell in the milder category 1 and only 8.2% was grouped under category 4 **(Fig. S2A**).

A high percentage of lethality was observed in both scramble and *rpgrip1l* morphants injected with 5 ng/nL, suggesting that excessive amounts of oligonucleotides may be associated with toxicity. Of note, unexpectedly also 2.5 ng/nL scramble injection caused severe phenotype underlining the relevance to performed MO and control injections for a certain gene at the same time to avoid artifacts due to injection-related factors such as volume, concentration, and embryo sensitivity. In a dose dependent manner, the percentage of fish falling in the more severe categories (4 and 3) was high (100% and 80.7% in the 5 ng/nL and 2.5 ng/nL group, respectively). Therefore, the MO doses were lowered to 1.25 and 0.5 ng/nL. In both cases a similar percentage of lethality occurred (48% with 1.25 ng/nL and 51% with 0.5 ng/nL), significantly lower than the previous tested concentration. The surviving embryos presented high deformity at 1.25 ng/nL (33% category 3, 11% category 2 and 7.4% category 1) and a milder phenotype at the lower dose of 0.5 ng/nL (8.8% category 3, 22% category 2, 12% category 1 and also 6.6% category 0) (**Fig. S2B)**.

Cilia whole-mount immunofluorescence staining with an anti-acetylated tubulin antibody was employed (**Fig. S2**). A complete loss of cilia was observed in *rpgrip1l*-MO, whereas *tmem67* morphants exhibited a significantly reduced area of cilia (**Fig. S2C,D)**. Consistently, the ciliary area of the Olfactory Placode (OP) of *tmem67* morphants was significantly smaller compared to scramble injected animals (**Fig. S2D,E**).

**Specificity of WT mRNA rescue**

To exclude the possibility that the observed rescue phenotype was due to *AHI1* WT mRNA overexpression, control injections were performed using *AHI1* WT mRNA alone (100 pg). Embryos injected only with *AHI1* mRNA were compared with not injected embryos and the morphological analysis of the phenotypic categories did not reveal significant differences. These results indicate that *AHI1* mRNA overexpression did not affect the phenotype (**Fig. S3A**).

To further evaluate the specificity of *ahi1* rescue observed after WT *AHI1* mRNA injection simultaneously with *ahi1*-MO, we performed an additional control experiment by co-injecting *AHI1* WT mRNA simultaneously with *rpgrip1l*-MO. In contrast to the rescue obtained in *ahi1* morphants, no phenotype rescue was detectable in *rpgrip1l* morphants. Moreover, embryos co-injected with *AHI1* WT mRNA and *rpgrip1l*-MO displayed a more severe phenotype compared with only *rpgrip1l*-MO injected embryos (**Fig. S3B**).

These findings support the conclusion that the rescue observed in *ahi1* morphants is specifically attributable to the restoration of *ahi1* function.

1. **FIGURES AND TABLE**


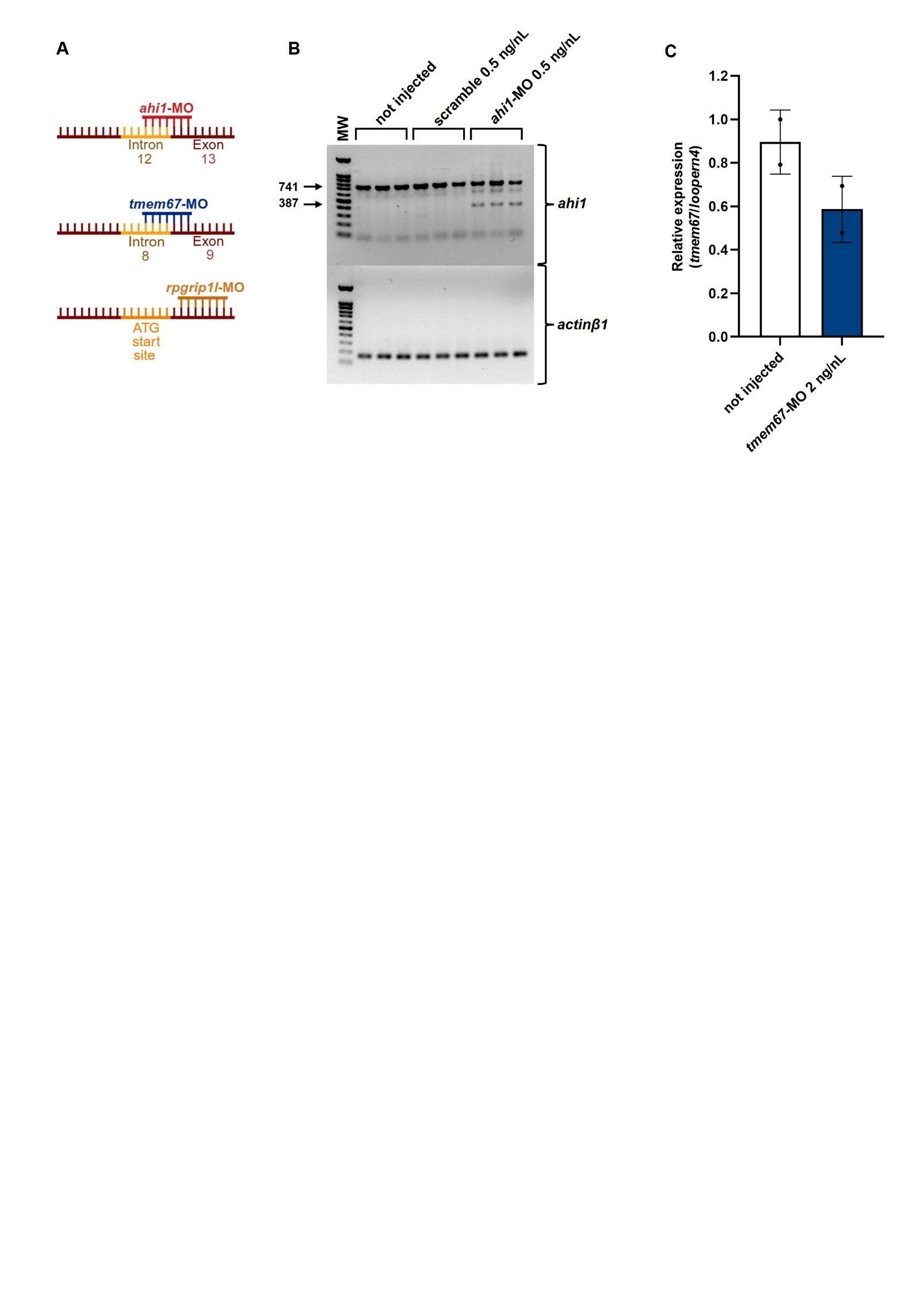


**Figure S1. *ahi1*, *tmem67* and *rpgrip1l* downregulation.** (A) *ahi1*, *tmem67* and *rpgrip1l* MOs target sites. (B-C) To determine the molecular effect of morpholino (MO) knockdown, the expression of *ahi1* and *tmem67* was evaluated in the RNA extracted from 2 dpf zebrafish control and morphants. (B) The splice-blocking *ahi1*-MO (0.5 ng/nL) targets the junction region between intron 12 and exon 13 of zebrafish *ahi1*, generating exon skipping and resulting in a 354 bp deletion [5]. RT-PCR amplification products showed two bands of, respectively, 741 bp and 387 bp. Based on band intensity quantitation the semiquantitative RT-PCR showed approximately 34.2% of the whole *ahi1* transcript was truncated, resulting in a shorter 354 bp amplicon (C) Splice-blocking *tmem67*-MO (2 ng/nL) targets the splice acceptor site between intron 8 and exon 9 [2]. qPCR analysis allowed to detect 34.5% reduction of *tmem67* expression in injected embryos compared to controls.


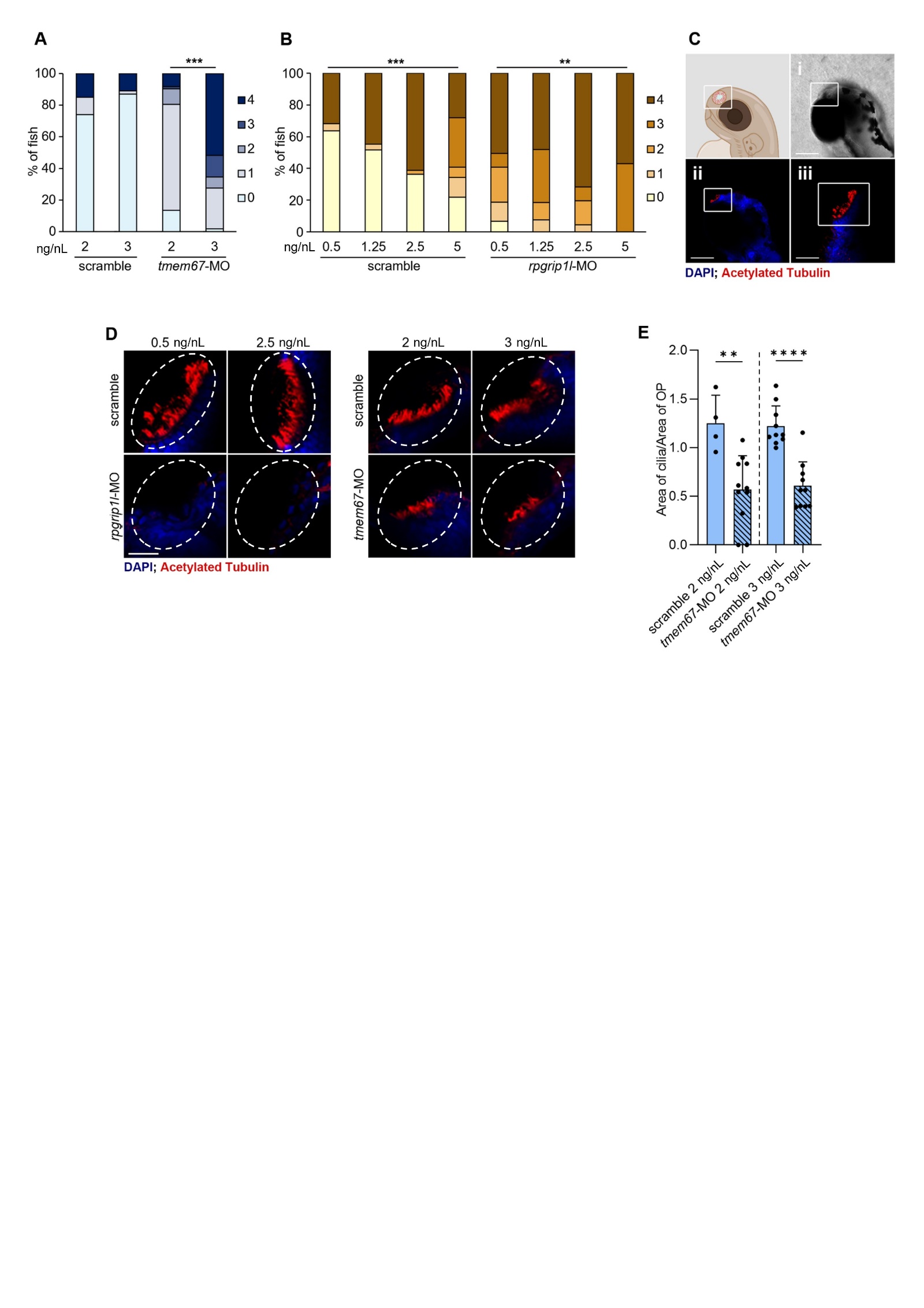
**Figure S2. Overview of the zebrafish olfactory placode (OP) and *tmem67* and *rpgrip1l* morphants characterization.** (A) Distribution of severity categories of *tmem67* morphants injected with two different MO concentrations (2 ng/nL and 3 ng/nL). A scramble oligonucleotide was used as control, and it was injected at all MO tested doses. (B) Distribution of severity categories of *rpgrip1l* morphants injected with four different concentrations (0.5 ng/nL, 1.25 ng/nL, 2.5 ng/nL and 5 ng/nL). A scramble oligonucleotide was used as control, and it was injected at all MO tested doses. (C) Scheme of a zebrafish scramble olfactory placode (OP). (i) Representative bright-field image of zebrafish OP, Magnification 25X, zoom 1. Scalebar: 150 µm. (ii) Representative whole mount immunofluorescence image of cilia in the OP stained with anti-acetylated tubulin antibody. Magnification 25X, zoom 1. Scalebar: 150 µm. (iii) Representative image of whole mount immunofluorescence of cilia in the OP, cilia stained with anti-acetylated tubulin antibody. Magnification 25X, zoom 3X. Scalebar: 50 µm. (D) Representative images of cilia area in OP (dashed line) in morphants injected with scramble (n≥4), *rpgrip1l*-MO (n≥6) and *tmem67*-MO (n≥11) for each concentration. Magnification 25X, zoom 3X. Scalebar: 25 µm. All the immunofluorescence images were acquired by confocal microscopy with water objective. Nuclei are stained with DAPI (in blue), cilia are stained by anti- acetylated Tubulin (in red). (E) Quantitation of cilia area in OP in *tmem67* morphants compared to scramble control at all concentrations. (** p ≤ 0.01, *** p ≤ 0.001, **** p ≤ 0.0001).


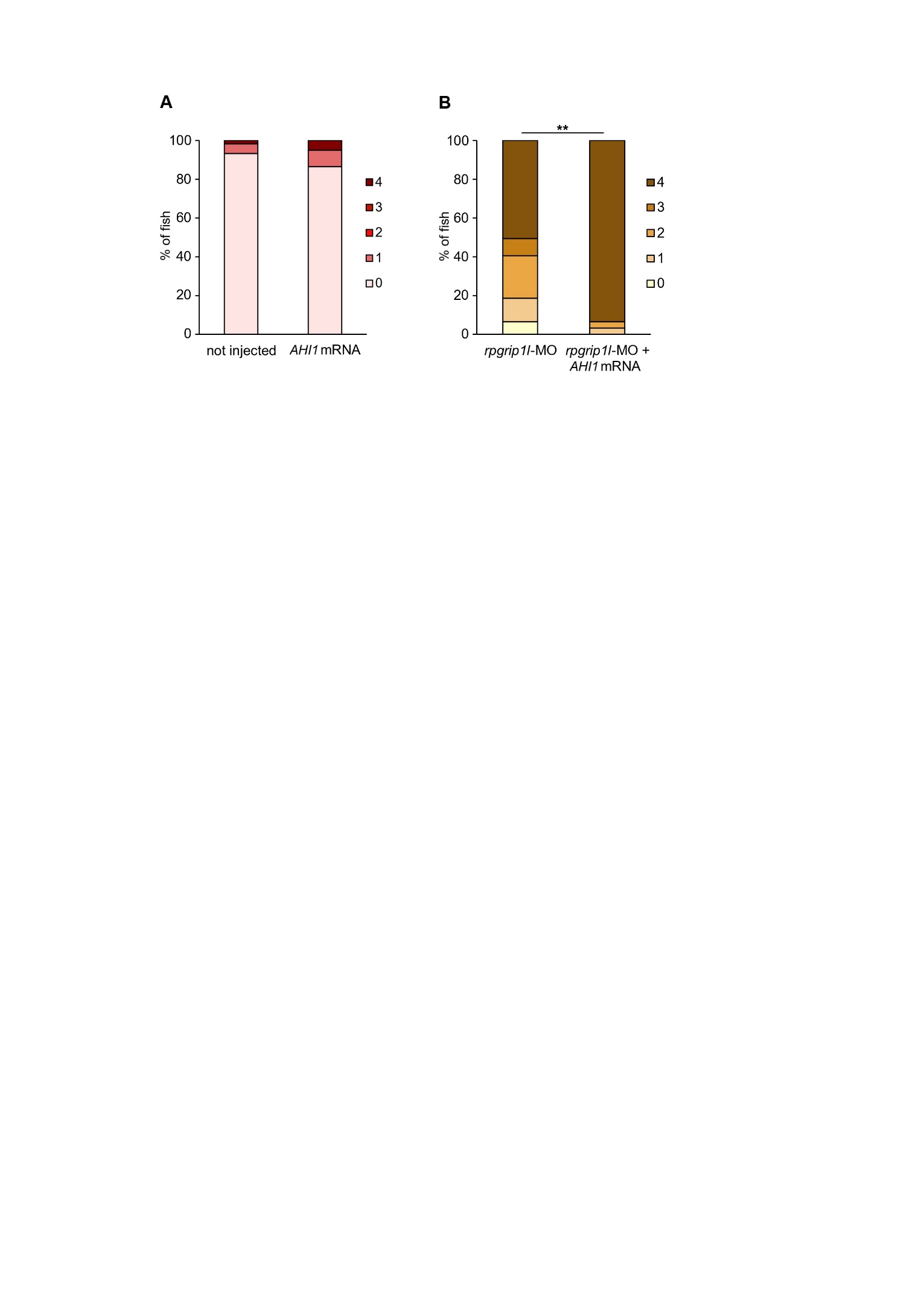


**Figure S3. Specificity of rescue. (**A) Distribution of severity categories of *AHI1* mRNA (100 pg) injected embryos compared to not injected control. (B) Distribution of severity categories of *rpgrip1l* morphants (0.5 ng/nL) coinjected with *AHI1* mRNA (100 pg). Embryos injected only with *rpgrip1l*-MO (0.5 ng/nL) were used as control.

**Table S1. Number of samples per experiment**

| **Morphological analysis** | | |  | **Immunofluorescence for olfactory placode** | | |
| --- | --- | --- | --- | --- | --- | --- |
| **Fig. 1B** | scramble 0.5 ng/nL | 19 |  | **Fig. 1C** | scramble 0.5 ng/nL | 3 |
|  | scramble 2 ng/nL | 18 |  |  | scramble 2 ng/nL | 4 |
|  | *ahi1*-MO 0.5 ng/nL | 15 |  |  | *ahi1*-MO 0.5 ng/nL | 4 |
|  | *ahi1*-MO 2 ng/nL | 11 |  |  | *ahi1*-MO 2 ng/nL | 4 |
| **Fig. 1D** | *ahi1*-MO | 154 |  | **Fig. 1E,F** | *ahi1*-MO | 10 |
|  | *ahi1*-MO + *AHI1* mRNA | 104 |  |  | *ahi1*-MO + PV *AHI1* mRNA | 8 |
|  | *ahi1*-MO + PV *AHI1* mRNA | 44 |  |  | *ahi1*-MO + *AHI1* mRNA - 3 | 4 |
|  | *ahi1*-MO + VUS1 *AHI1* mRNA | 82 |  |  | *ahi1*-MO + *AHI1* mRNA - 2 | 3 |
|  | *ahi1*-MO + VUS2 *AHI1* mRNA | 39 |  |  | *ahi1*-MO + *AHI1* mRNA - 1 | 4 |
| **Fig. S2A** | scramble 2 ng/nL | 100 |  |  | *ahi1*-MO + *AHI1* mRNA - 0 | 3 |
|  | scramble 3 ng/nL | 46 |  |  | *ahi1*-MO + VUS1 *AHI1* mRNA - 3 | 4 |
|  | *tmem67*-MO 2 ng/nL | 134 |  |  | *ahi1*-MO + VUS1 *AHI1* mRNA - 2 | 4 |
|  | *tmem67*-MO 3 ng/nL | 58 |  |  | *ahi1*-MO + VUS1 *AHI1* mRNA - 1 | 4 |
| **Fig. S2B** | scramble 0.5 ng/nL | 22 |  |  | *ahi1*-MO + VUS1 *AHI1* mRNA - 0 | 3 |
|  | scramble 1.25 ng/nL | 29 |  | **Fig. 1G** | *ahi1*-MO | 3 |
|  | scramble 2.5 ng/nL | 44 |  |  | *ahi1*-MO + VUS2 *AHI1* mRNA | 3 |
|  | scramble 5 ng/nL | 32 |  | **Fig. S2D,E** | scramble 2 ng/nL | 5 |
|  | *rpgrip1l*-MO 0.5 ng/nL | 91 |  |  | scramble 3 ng/nL | 10 |
|  | *rpgrip1l*-MO 1.25 ng/nL | 54 |  |  | *tmem67*-MO 2 ng/nL | 11 |
|  | *rpgrip1l*-MO 2.5 ng/nL | 46 |  |  | *tmem67*-MO 3 ng/nL | 12 |
|  | *rpgrip1l*-MO 5 ng/nL | 10 |  |  | scramble 0.5 ng/nL | 4 |
| **Fig. S3A** | not injected | 60 |  |  | scramble 2.5 ng/nL | 6 |
|  | *AHI1* mRNA | 60 |  |  | *rpgrip1l*-MO 0.5 ng/nL | 6 |
| **Fig. S3B** | *rpgrip1l*-MO 0.5 ng/nL | 91 |  |  | *rpgrip1l*-MO 2.5 ng/nL | 7 |
|  | *rpgrip1l*-MO 0.5 ng/nL + *AHI1* mRNA | 30 |  |  |  |  |

**Table S2. *In silico* predictions of selected variants.** Variants pathogenicity was evaluated using the dbNSFP v5.0 ([https://www.dbnsfp.org](https://www.dbnsfp.org/)) [7], a widely used database developed for functional prediction and annotation of all potential non-synonymous single-nucleotide variants (nsSNVs) in the human genome. It reports pathogenicity based on prediction scores from several algorithms.

| **Tools** | **c.2168 G>A** (**R723Q)**  **PV**  **ACMG:8** | **c.2273 A>C** (**H758P)**  **VUS1**  **ACMG:3** | **c.2009 T>C** (**L670P)**  **VUS2**  **ACMG:4** |
| --- | --- | --- | --- |
| **SIFT_score** | 0,037 (D) | 0,053 (T) | 0,001 (D) |
| **Polyphen2_ HDIV_score** | 1 (D) | 0,999 (D) | 1 (D) |
| **Polyphen2_ HVAR_score** | 0,971 (D) | 0,998 (D) | 0,99 (D) |
| **PROVEAN_score** | -3,42 (D) | -2,94 (D) | -5,46 (D) |
| **MetaSVM_score** | 0,1805 (D) | -0,6167 (T) | 0,0808 (D) |
| **MetaLR_score** | 0,5453 (D) | 0,26 (T) | 0,4558 (T) |
| **M-CAP_score** | 0,04377 (D) | 0,030929 (D) | 0,177858 (D) |
| **MVP_score** | 0,88670 (D) | 0,693340 (T) | 0,88696 (D) |
| **CADD_phred** | 33 (D) | 26,7 (D) | 31 (D) |

D= deleterious; T=Tolerated

**References**

1. Simms, R.J., et al., *Modelling a ciliopathy: Ahi1 knockdown in model systems reveals an essential role in brain, retinal, and renal development.* Cell Mol Life Sci, 2012. **69**(6): p. 993–1009.

2. Leightner, A.C., et al., *The Meckel syndrome protein meckelin (TMEM67) is a key regulator of cilia function but is not required for tissue planar polarity.* Hum Mol Genet, 2013. **22**(10): p. 2024–40.

3. Mahuzier, A., et al., *Dishevelled stabilization by the ciliopathy protein Rpgrip1l is essential for planar cell polarity.* J Cell Biol, 2012. **198**(5): p. 927–40.

4. Schindelin, J., et al., *Fiji: an open-source platform for biological-image analysis.* Nat Methods, 2012. **9**(7): p. 676–82.

5. Zhu, L., et al., *Mutant Ahi1 Affects Retinal Axon Projection in Zebrafish via Toxic Gain of Function.* Front Cell Neurosci, 2019. **13**: p. 81.

6. Vanhauwaert, S., et al., *Expressed repeat elements improve RT-qPCR normalization across a wide range of zebrafish gene expression studies.* PLoS One, 2014. **9**(10): p. e109091.

7. Liu, X., et al., *dbNSFP v3.0: A One-Stop Database of Functional Predictions and Annotations for Human Nonsynonymous and Splice-Site SNVs.* Hum Mutat, 2016. **37**(3): p. 235–41.
